# Resistance to Somatostatin Analogs in Italian Acromegaly Patients: The MISS Study

**DOI:** 10.3390/jcm12010025

**Published:** 2022-12-20

**Authors:** Alessandro Maria Berton, Nunzia Prencipe, Luca Bertero, Marco Baldi, Chiara Bima, Marina Corsico, Antonio Bianchi, Giovanna Mantovani, Francesco Ferraù, Paola Sartorato, Irene Gagliardi, Ezio Ghigo, Silvia Grottoli

**Affiliations:** 1 Division of Endocrinology, Diabetology and Metabolism, Department of Medical Sciences, University of Turin, 10126 Turin, Italy; 2 Pathology Division 2, Department of Medical Sciences, University of Turin, 10126 Turin, Italy; 3 Department of Translational Medicine and Surgery, University Cattolica del Sacro Cuore, Endocrinology and Diabetology Unit, Fondazione Policlinico Universitario A. Gemelli, IRCCS, 00168 Rome, Italy; 4 Department of Neurosciences, University of Turin, 10126 Turin, Italy; 5 Fondazione IRCCS Ca’ Granda Ospedale Maggiore Policlinico, Endocrinology Unit, 20122 Milan, Italy; 6 Department of Clinical Sciences and Community Health, University of Milan, 20122 Milan, Italy; 7 Department of Human Pathology of Adulthood and Childhood G. Barresi, University of Messina, Endocrine Unit, University Hospital G. Martino, 98125 Messina, Italy; 8 Internal Medicine 2, Ca’ Foncello Hospital, AULSS2, 31100 Treviso, Italy; 9 Section of Endocrinology and Internal Medicine, Department of Medical Sciences, University of Ferrara, 44100 Ferrara, Italy

**Keywords:** first-generation somatostatin receptor ligands, growth hormone-secreting adenoma, SSTR2, CAM5.2 granulation pattern, magnetic resonance imaging, precision medicine

## Abstract

Approximately 60% of acromegaly patients are not adequately controlled by first-generation somatostatin receptor ligands. This multicenter retrospective study aimed to identify the most relevant biomarkers specific for the Italian acromegaly population. Resistant patients were enrolled consecutively based on time of neurosurgery, while responders were collected in a 1:2 ratio. Clinical characteristics and T2-intensity on MRI scans at diagnosis were retrospectively re-evaluated. Histological analyses of CAM5.2 granulation patterns and SSTR2 expression were centrally performed. Sixty-three resistant patients and thirty-three responders were enrolled. A low-grade SSTR2 expression was the most relevant predictor of resistance identified (OR 4.58, *p* = 0.013), even considering CAM5.2 immunohistochemistry (OR 2.65, *p* = 0.047). T2-iso/hyperintense pattern on MRI was also associated with a 3.3-fold greater probability of poor response to medical treatment (*p* = 0.027), as well as a young age at diagnosis (OR 0.96, *p* = 0.035). In those patients treated only after neurosurgery due to persistent GH-hypersecretion (51, 53.1%) the absence of any appreciable adenomatous remnant on postoperative MRI was associated with a negligible risk of resistance (OR 0.04, *p* = 0.003). In the Italian acromegaly population, a low-grade SSTR2 expression seems to be the most relevant predictor of resistance to first-generation somatostatin receptor ligands, followed by a SG/intermediate cytokeratin pattern and a T2-iso/hyperintense MRI signal.

## 1. Introduction

In acromegaly, first-generation somatostatin receptor ligands (fg-SRL) are the treatment of choice in case of post-surgical persistence of growth hormone (GH) hypersecretion [1,2,3,4]. Furthermore, fg-SRL can be effectively used as a first therapeutic approach for neoadjuvant purposes to reduce the size of pituitary adenomas if neurosurgery is unlikely to be curative, or when it is refused or contraindicated [4,5,6]. 

Nonetheless, approximately 60% of patients do not achieve adequate disease control during fg-SRL [4,7,8,9]. In cases of complete resistance, both age-adjusted insulin-like growth factor I (IGF-I) and random GH (r-GH) levels remain elevated in the absence of a significant reduction of tumor volume (greater than or equal to 20%), even after 6 months of treatment with full-dose fg-SRL [8,10,11]. Otherwise, in case of partial resistance, a discrepancy between IGF-I and GH levels may be recorded.

Since current medical treatment for acromegaly is still based on a trial and error approach [5,9,12], the identification of response biomarkers would facilitate decision making by allowing the appropriate drug to be chosen for each patient at the right time, thus improving the response rate and achieving disease control as quickly as possible [9].

The main clinical predictors of fg-SRL resistance recognized in the literature include: male sex [5,13,14], young age [5,13,14,15,16,17] and tumor hyperintensity on T2-weighted magnetic resonance imaging (MRI) scans [1,7,13,15,16]; however, IGF-I and r-GH values at diagnosis also deserve to be taken into consideration [5,8,13,14,15,16,17]. Furthermore, the most relevant histological and molecular characteristics identified so far are the following: the grade of expression of the type 2 somatostatin receptors (SSTR2) [1,7,8,15,18], the pathological classification based on cytoplasmic granulation and keratin pattern analyzed by the CAM5.2 antibody [1,7,8,15], the proliferation index Ki-67 [7,18,19] and the presence of aryl hydrocarbon receptor-interacting protein (AIP) gene mutation [8,20,21]. Some of these features appear to be attributable to genetic and epigenetic modifications specific to the patient’s population or influenced by the environment of the country of origin [20,22]. It could, therefore. be assumed that at least some of these factors may be more relevant in local communities.

The purpose of this clinical study was to identify the most relevant predictors of resistance to fg-SRL among those widely available in the clinical practice of Tertiary Referral centers, specific for the Italian acromegaly population.

## 2. Materials and Methods

The MISS (MRI and histological features as predictors of response to treatment with first-generation somatostatin receptor ligands) study was an Italian, retrospective clinical research involving the referral centers of Turin, Rome, Milan, Messina, Treviso and Ferrara. Study duration from 5/2018 to 12/2020.

### 2.1. Study Population and fg-SRL Resistance Assessment

Patients resistant to fg-SRL were selected at the respective participating centers based on the temporal criterion, starting with those who most recently underwent neurosurgical intervention (NSI). Responders were then collected in a 1:2 ratio with respect to resistant cases, identifying fg-SRL sensitive patients with NSI performed at the closest date to that of resistant subjects. As a proof-of-concept study, it was not possible to perform a calculation for the statistical power of the samples. Therefore, the recruitment process aimed to obtain an overall sample of almost 100 patients, representative of the Italian acromegaly population.

Inclusion criteria were as follows: (1) age 18-85 years; (2) GH-secreting pituitary adenoma; (3) previous NSI with availability of histological samples; (4) biochemical evaluation of fg-SRL resistance (i.e., octreotide LAR or lanreotide autogel); (5) written informed consent.

Exclusion criteria were: (1) recent pregnancy or breastfeeding; (2) radiotherapy treatment before NSI or in the following 9 months (unless the diagnosis of fg-SRL resistance was made before NSI); (3) concomitant treatment with dopamine agonists or pegvisomant in the 6-month assessment of fg-SRL resistance.

Failure to respond to medical treatment was defined by the presence of uncontrolled age-adjusted IGF-I after 6 months of full-dose fg-SRL, as a neoadjuvant or adjuvant approach to NSI [10,11]. Indeed, although r-GH levels were also taken into account to evaluate the response to fg-SRL, in case of discrepant results between the two parameters, more importance was given to age-adjusted IGF-I, in consideration of the its greater stability. Serum GH (ng/mL) and IGF-I levels (ng/mL) were measured at the University of Turin in duplicate by IRMA and RIA, respectively. Instead, both analytes were assayed with chemiluminescence methods in the other centers (Table S1). Assay names and their manufacturers, together with all available data regarding the sensitivity limits, the inter and intra-assay coefficients of variation, as well as the standards used for calibration are given in Table S1. IGF-I upper limit of normal (ULN) was taken into consideration to reduce variability between different immunoassays.

In addition, all available pituitary MRI performed at diagnosis, three months postoperatively, and finally, after 6 months of full-dose fg-SRL were thoroughly reevaluated. In particular, a possible tumor shrinkage of less than 20% or an increase in its volume during full-dose fg-SRL was investigated and recorded.

### 2.2. Histological Analysis

Hematoxylin and eosin histological slides were reviewed, as well as immunohistochemistry (IHC) for GH, to confirm the diagnosis of somatotroph adenoma. IHC for prolactin (PRL) was also performed. 

**Figure 1:**
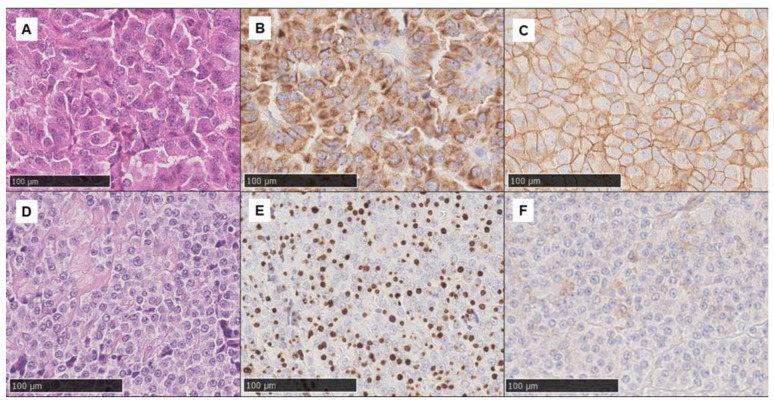
Histological images showing two example cases (original magnification 200×). The first set (**A**–**C**) shows a somatotroph adenoma (**A**: hematoxylin and eosin) with a densely granulated CAM5.2 pattern (**B**) and diffuse SSTR2 positivity (score 3) (**C**). Conversely, the second set (**D**–**F**) shows a somatotroph adenoma (**D**: hematoxylin and eosin) sparsely granulated according to the extensive dot-like CAM5.2 pattern (**E**) and with focal, mainly cytoplasmic, SSTR2 positivity (score 1) (**F**).

Proliferation activity was assessed by Ki-67 IHC, while p53 positivity was evaluated as the rate of positive cells. IHC for CAM5.2 (Roche—Ventana Medical Systems, Oro Valley, AZ, USA) was performed to ascertain adenoma subtype using a ≥70% cut-off (i.e., if perinuclear cytoplasmic or dot-like staining was observed in ≥70% of cells, the densely or sparsely granulated subtype was assigned, respectively; conversely, if no pattern was present in ≥70% of cells, the adenoma was classified as intermediate) (Figure 1). SSTR2 IHC (clone UMB1, Abcam, Cambridge, UK) was scored as follows: 0: negative; 1: cytoplasmic stain or membranous <25% cells; 2: membranous 25–75% of cells: 3: membranous >75% of cells (Figure 1). All IHC analyses were performed centrally at the University Hospitals of Turin and Rome.

### 2.3. Radiological Re-Evaluation

**Figure 2:**
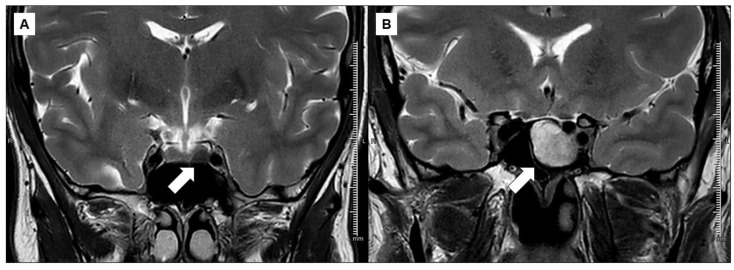
Radiological images showing two example cases. Left a markedly hypointense somatotroph adenoma in T2-weighted MRI scans (**A**). By way of comparison, a hyperintense somatotroph adenoma in T2-weighted MRI scans (**B**) is shown on the right.

The neuroradiological characteristics of high resolution (1.5 or 3 T) MRI were re-evaluated at each recruiting center by the same radiologist. T2-weighted MRI signal of the GH-secreting adenoma was defined by comparing the intensity of the tumor with that of normal pituitary tissue and, when this was not visible, to the grey matter of the temporal lobe [23] (Figure 2). 

### 2.4. Statistical Analysis

Continuous variables were expressed as mean and standard deviation (SD) or median and interquartile range (IQR), depending on their distribution. Categorical variables were reported as number and percentage. Between-group comparisons for continuous variables were performed with the Student *t*-test or the Mann–Whitney U-test, where appropriate. Correlation between categorical variables were calculated by the Chi-square test or the Fisher’s exact test. Logistic regression models were used to describe the possible correlation between independent and dependent variables; the stepwise approach was used and the variables were not included in the model for *p* > 0.1. 

*p*-values < 0.05 were considered statistically significant; no corrections to *p*-values were made. A *p*-value < 0.1 was considered for the inclusion of variables in multivariable regression models. 

Statistical analysis was performed using MedCalcTM^®^ (version 18.11.3, MedCalc Software Ltd., Ostend, Belgium). Figures were made using GraphPad PrismTM^®^, version 8.01 (version 8.01, GraphPad Software LLC, San Diego, CA, USA).

## 3. Results

We enrolled 96 acromegaly patients divided in two group based on disease control after 6 months of full-dose fg-SRL. Consequently, 63 patients (65.6%) were defined as resistant to fg-SRL (R-SRL), while the remaining 33 (34.4%) patients as responders (S-SRL). Of 96 recruited patients, in 42 subjects who underwent NSI without neo-adjuvant treatment with fg-SRL, the resistance condition was assessed after surgery (28 R-SRL, 14 S-SRL). No significant differences were observed in the proportion of fg-SRL responders between the groups of patients who received the neoadjuvant medical approach and those treated only postoperatively.

**Figure 3:**
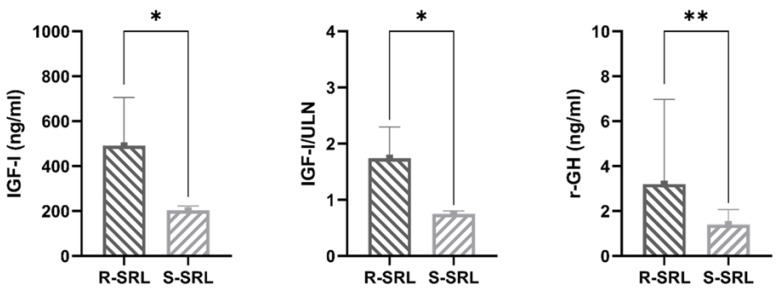
Median values of biochemical disease control parameters at enrolment in patients resistant (R-SRL) and in those responsive to somatostatin receptor ligands (S-SRL) (* *p* < 0.0001, ** *p* = 0.0002).

Median age-adjusted IGF-I (491 vs. 202.5 ng/mL, *p* < 0.0001), IGF-I/ULN (1.74 vs. 0.75, *p* < 0.0001) and r-GH values (3.2 vs. 1.4 ng/mL, *p* = 0.0002) during fg-SRL treatment provided significantly different results between the two comparison groups, thus ensuring the correct identification of R-SRL (Figure 3). Of note, r-GH evaluation was available only in 76% of patients (Table 1).

The overall volumetric response to fg-SRL, retrospectively re-evaluated in 66.7% of patients, was poor and clearly less relevant among R-SRL than in S-SRL (0 vs. 20%, respectively), although not statistically significant (Table 1). There was no significant difference in the prevalence of fg-SRL resistance between sexes (females 58.7 vs. 66.7%, R-SRL vs. S-SRL), nor between the two drugs administered: octreotide LAR (48.4 vs. 53.3%) or lanreotide autogel (51.6 vs. 46.7%) (Table 1).

### 3.1. Patients’ Characteristics at Diagnosis

Among the clinical variables analyzed at the time of diagnosis (Table 2), the age of R-SRL was lower, with a mean difference of 5.5 years (41.6 vs. 47.1 years, R-SRL vs. S-SRL, *p* = 0.052). Furthermore, mean age-adjusted IGF-I levels were higher in this patient group (905.7 vs. 779.4 ng/mL, *p* = 0.055), in contrast with both the remaining biochemical parameters of disease activity (i.e., IGF-I/ULN and r-GH) and median PRL levels (Table 2). 

In a multivariate logistic regression model, age at diagnosis was significantly associated with the condition of resistance to fg-SRL (OR 0.96, AUC 0.62, *p* = 0.035), even when corrected for IGF-I levels (Table 3).

Resistant tumors showed a significantly larger maximum diameter with a median difference of 5 mm (20 vs. 15 mm, R-SRL vs. S-SRL, *p* = 0.049) (Table 2) and an iso/hyperintense signal in T2-weighted MRI scans was more frequent among them (75 vs. 47.6%, *p* = 0.048). Instead, no significant difference was found in tumor invasiveness as evaluated on MRI. 

Note that the MISS study population included fewer micro- than macroadenomas (8.4 vs. 91.6%), homogeneously between R-SRL and S-SRL. More importantly, a thorough re-evaluation of T2 signal intensity was possible at the respective recruiting centers in only 76% of subjects (Table 2, Table 3 and Table 4).

In a multivariate regression model, T2-iso/hyperintensity of the tumor resulted in the strongest radiological predictor of resistance among those analyzed (OR 3.3, AUC 0.64, *p* = 0.027), even considering maximal diameter for inclusion in the model (Table 3). 

### 3.2. Histological and Molecular Features

Among GH-secreting tumors, 37.5% had a positive IHC for PRL, with no significant differences between resistant and responding patients to fg-SRL.

As expected, we confirmed that low-grade SSTR2 expression (i.e., score 0–1 vs. 2–3) was significantly more frequent among resistant adenomas (40.9 vs. 14.3%, R-SRL vs. S-SRL, *p* = 0.024) (Table 5). Instead, IHC for CAM5.2 showed only a slightly higher prevalence of an SG/intermediate granulation pattern among R-SRL (respectively 65.1 vs. 34.9%, SG/intermediate vs. DG, *p* = 0.102); while the two phenotypes were equally distributed among S-SRL (45.4 vs. 54.5%). Finally, neither the Ki-67 proliferation index nor the p53 positivity provided significantly different results between the two comparison groups (Table 5).

It should be noted that the grade of SSTR2 expression was significantly different between patients pretreated with fg-SRL or not (*p* < 0.001); in particular, elevated expression of SSTR2 (grade 2-3) was present in 84.4% of treatment-naïve patients, but only in 48.78% of those who received a full-dose of fg-SRL before NSI. However, in multivariate analysis, low SSTR2 expression remained a significant predictor of fg-SRL resistance (OR 5.09, *p* = 0.015, AUC 0.6) regardless of whether medical treatment was performed in the pre- or post-surgery (variable not included in the model for *p* > 0.1). 

In the multivariate regression model, both low-grade SSTR2 expression (OR 4.58, *p* = 0.013) and a SG/intermediate granulation pattern proved to be significant predictors of resistance to fg-SRL (OR 2.65, *p* = 0.047, AUC 0.7), with greater importance attributable to the former (Table 3). 

Finally, by comparing the predictive role of the cytokeratin staining pattern versus T2-intensity on MRI scans, in the subgroup of patients in which this re-evaluation was performed, CAM5.2 IHC achieved better results (OR 5.56, *p* = 0.003, vs. OR 3.24, *p* = 0.045, AUC 0.76) (Table 3). 

### 3.3. Post-Operative Characteristics in NSI Patients without Neo-Adjuvant Treatment 

**Figure 4:**
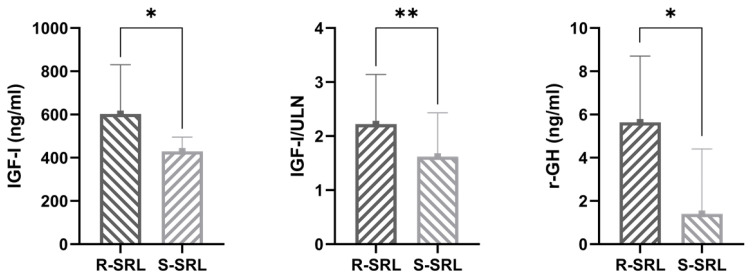
Median values of biochemical disease control parameters three months after surgery in patients resistant (R-SRL) and in those responsive to somatostatin receptor ligands (S-SRL) (* *p* < 0.01, ** *p* = 0.02).

In the end, we searched for any further promising predictors of fg-SRL resistance at the routine three months post-operative re-evaluation. For this analysis, we selected only those 42 patients (28 R-SRL, 14 S-SRL) who underwent NSI without neo-adjuvant treatment with fg-SRL. In this case, both the absence of post-operative appreciable remnant on MRI (17.2 vs. 72.2%, R-SRL vs. S-SRL, *p* = 0.0005) and lower biochemical parameters of disease activity (IGF-I 603 vs. 429.5 ng/mL, *p* = 0.002; IGF-I/ULN 2.22 vs. 1.62, *p* = 0.02; r-GH levels 5.64 vs. 1.4 ng/mL, *p* = 0.003) were associated with a subsequent good response to fg-SRL (Figure 4). Among these variables, the absence of any appreciable post-operative remnant on MRI re-evaluation lead to a 0.04-fold chance of being resistant (OR 0.04, AUC 0.82, *p* = 0.0003), even considering r-GH for the inclusion in the model (Table 3).

## 4. Discussion

The MISS study confirms the presence of multiple reliable predictors of resistance to fg-SRL in a representative cohort of Italian acromegaly patients.

As expected, the most important among them are the IHC features of GH-secreting tumors, mainly represented by a low-grade SSTR2 expression and an SG/intermediate granulation pattern. Furthermore, among the patients’ clinical characteristics, age at diagnosis is associated with a 0.96-fold lower probability of resistance for each year of age. Finally, the iso/hyperintense aspect of somatotroph adenomas on T2-weighted MRI scans represents an additional reliable and readily available response biomarker.

Our data are consistent with those provided by the literature [1,7,8,13,15,16,17,18], but also confer a specific weight to each predictor, thanks to the use of the multivariate regression analyses. Indeed, at first, we identified the most promising variables among all the clinical, radiological and histological characteristics considered separately (Table 2 and Table 5). Then, once confirmed significant correlations with the response to fg-SRL (Table 4), we compared the relevant predictors in multivariate regression models, thus obtaining a specific coefficient for each one (Table 3). 

In our results, the presence of a low-grade SSTR2 expression appears to be the most relevant predictor of resistance to fg-SRL; associated with a 4.58-fold higher risk, even considering the CAM5.2 IHC (Table 3). As known, high SSTR2 levels are essential for responsiveness to the fg-SRL, which mainly act by binding and activating this specific receptor subtype, and less to SSTR5 [5,24]. However, this mechanism alone appears to be insufficient [8,15], as approximately half of clinically resistant cases exhibit a high-grade SSTR2 expression phenotype. It is agreed that in most of these subjects, the poor response is due to a low expression of AIP, in turn deeply involved in the transduction of the intracellular signal of SSTR [8,21]. It should be noted that several potential scoring systems for IHC assessment of SSTR2 expression have been proposed over the years [25]. In the MISS study, an attempt was made to apply a score that was easily reproducible and did not take into account staining intensity, also to facilitate inter-observer reproducibility. However, considering the very recent data of Ilie et al. [26] in support of the immunoreactivity score (IRS) as a preferential scoring system, a consensus aimed at defining a standardized and shared score for the evaluation of SSTR2 would be useful [25].

However, the tumor cellular ultrastructure may play a role [5]. Indeed, cytokeratins are structural components of the cell providing mechanical stability and probably participate in mechanisms of exocytosis and receptor signal transduction [27]. Previous studies described greater efficacy of fg-SRL in DG tumors with higher response rate and a greater reduction in IGF-I levels [28,29]; this observation is, in turn, consistent with the demonstration of a poor SSTR2 representation on cells of somatotroph adenoma with a dot-like cytokeratin pattern [27]. Finally, a lower expression of E-cadherin by SG tumors, a component of the adherent junction, could be partially responsible for the increase of their proliferative and invasive properties [29]. Intriguingly, GH-secreting tumors expressing low levels of E-cadherin show a worse response to fg-SRL treatment, probably partly independent of the degree of SSTR2 expression [30]. Indeed, a clear association between the IHC E-cadherin score and the grade of SSTR2 expression has only occasionally been found in previous studies [30,31]. Conversely, low E-cadherin levels were sometimes associated with poor response to fg-SRL, even in patients with high SSTR2 levels. Therefore, E-cadherin and SSTR2 are likely to represent two parallel but independent regulators of response to fg-SRL. 

In the MISS study, the sparse granulation pattern conferred a clearly less relevant risk of being resistant to fg-SRL compared to lower SSTR2 expression, although it is still significant (Table 3 and Figure 4). Of note, in our analysis, tumors with an intermediate pattern resembled those SG in their clinical behavior, although in past series in which three morphological phenotypes (i.e., SG, DG and intermediate pattern) have been reported, the transitional group has sometimes shown a better response to medical treatment than SG [1,28,29]. 

On the other hand, although highly informative, the histological features of somatotroph tumors are available only in the postoperative period. In contrast, MRI is, today, the most widely used imaging technique for both diagnosis and follow-up of hypothalamic-pituitary lesions.

In this context, our analyses confirm that T2-weighted MRI scans are useful for promptly identifying, at diagnosis, adenomas least susceptible to medical treatment with fg-SRL. In fact, albeit to a lesser extent than the previous parameters, the T2-iso/hyperintense pattern is also associated with a 3.3-fold greater probability of resistance to medical treatment (Table 3 and Table 4). GH-secreting T2-hypointense adenomas had already been reported as smaller and less invasive (exceptionally involving the cavernous sinus), but associated with higher IGF-I and GH levels at diagnosis [23]. More importantly, T2-hypointensity predicted both improved biochemical and morphological response to fg-SRL [32,33,34]. Unfortunately, today there is no clear explanation for the existing correlation between the appearance of T2-hypointense somatotroph adenomas and their susceptibility to treatment with fg-SRL. In fact, although several authors described a correlation between a lower T2-intensity and a DG cytokeratin phenotype [34,35], as well as between cell tumor ultrastructure and SSTR expression [36], evidence is still scant and often conflicting [23,33,36]. 

Of note, in a subset analysis conducted only on those patients undergoing neurosurgery without any neoadjuvant medical treatment, the absence of a clearly appreciable adenomatous remnant on post-surgical MRI leads to a negligible probability of subsequent resistance to fg-SRL, even considering r-GH at three months postoperative for inclusion in the model (Table 3). These results can be easily explained by the significant reduction of tumor mass and in the number of adenomatous somatotroph cells obtained by NSI. Indeed, several previous studies have shown a greater response to fg-SLR after NSI [37,38,39,40]; but, in the MISS study, no direct comparison was made between pre- and postoperative response in the subgroup of patients already receiving fg-SRL as a neoadjuvant approach. However, when looking at the entire cohort, there was no difference in the proportion of responders between the neoadjuvant and adjuvant treatment groups.

The MISS study presents some limitations. First, the retrospective design; second, the inevitable heterogeneity in the assays used at the different participating centers for the determination of GH and IGF-I (Table S1); finally, the absence of a complete biochemical and neuroradiological re-evaluation for some of the patients recruited. Indeed, it should be noted that r-GH values were only available in 76% of cases (Table 1), as well as an accurate re-evaluation of the T2-intensity MRI signal of somatotroph adenomas (Table 2). In addition, although GH nadir values during the oral glucose tolerance tests were also collected during the study, the data were available for only about 50% of the patients; certainly too few to obtain reliable regression models.

Finally, the aim of the MISS study was to define the best predictors of resistance to fg-SRL among the most widely used clinical, radiological and histological ones. Therefore, a specific genetic panel (i.e., AIP mutation) or further IHC analyses to search for less frequently used histological predictors (i.e., E-cadherin, Filamin A, SSTR5) were never considered. 

## 5. Conclusions

Our data confirm the presence of reliable predictors of fg-SRL resistance (age at diagnosis, cytokeratin granulation pattern, SSTR2 expression, T2-intensity on MRI, post-operative remnant persistence) in the Italian acromegaly population. Clearly, all these parameters partially overlap, defining the fg-SRL response alone or in combination. However, when considered together in multivariate regression models, the grade of expression of SSTR2 is confirmed to be the most relevant, regardless of the cytokeratin phenotype. All these factors deserve to be evaluated before setting up medical treatment in acromegaly patients and future guidelines should consider this emerging evidence when making recommendations on therapeutic choice. Nonetheless, it would be necessary to collect all the data published in the major international studies to confirm the results obtained so far and to formulate even more precise multivariable predictive models before implementing acromegaly therapeutic algorithms on this basis.

## Figures and Tables

**Table 1:** Main characteristics of the acromegaly population (96 patients) at the recruitment.

Data Available (n, %)	Variable	All patient	R-SRL	S-SRL	*p*-Value
96, 100	Female (n, %)	59, 61.5	37, 58.7	22, 66.7	0.59
92, 95.8	Fg-SRL (n, %)				
	- Octreotide	46, 50	30, 48.4	16, 53.3	0.82
	- Lanreotide	46, 50	32, 51.6	14, 46.7	
92, 95.8	IGF-I (ng/mL)	394.5 [224–587.5]	491 [390–705]	202.5 [181–223]	**<0.0001**
92, 95.8	IGF-I/ULN	1.38 [0.83–1.91]	1.74 [1.36–2.3]	0.75 [0.61–0.8]	**<0.0001**
73, 76	r-GH (ng/mL)	2.4 [1.4–5.8]	3.2 [2–6.97]	1.4 [0.6–2.07]	**0.0002**
64, 66.7	TVR (%)	0 [0–25]	0 [0–21.25]	20 [0–34.64]	0.18

Abbreviations: R-SRL, patients resistant to somatostatin receptor ligands; S-SRL, patients responsive to somatostatin receptor ligands; fg-SRL, first-generation somatostatin receptor ligands; IGF-I, insulin-like growth factor I; r-GH, random growth hormone levels; TVR, total volume reduction.

**Table 2:** Clinical, biochemical and MRI radiological features at diagnosis.

Data Available (n, %)	Variable	All Patient	R-SRL	S-SRL	*p*-Value
96, 100	Age (years)	43.5 ± 13.3	41.6 ± 12.03	47.1 ± 14.9	0.052
94, 97.9	IGF-I (ng/mL)	862.7 ± 303.2	905.7 ± 326	779.4 ± 236.05	0.055
94, 97.9	IGF-I/ULN	3 ± 1.1	3.12 ± 1.12	2.79 ± 1	0.162
74, 77.1	r-GH (ng/mL)	11.99 [5.9–32.2]	13 [6.15–47.5]	8.81 [5–26.2]	0.257
89, 92.7	PRL (ng/mL)	13.5 [10–32.5]	13.3 [10–31.06]	18.1 [10–32.8]	0.593
95, 98.9	Maximal tumor diameter (mm)	18 [13–24.5]	20 [13–25.7]	15 [13.5–2]	**0.049**
95, 98.9	Microadenomas (n, %)	8, 8.4	6, 9.5	2, 6.2	0.879
91, 94.8	Cavernous sinus invasion (n, %)	57, 62.6	42, 68.8	15, 50	0.129
77, 80.2	Suprasellar extension (n, %)	49, 63.6	40, 67.8	9, 50	0.274
77, 80.2	Intrasellar extension (n, %)	26, 33.8	19, 32.7	7, 36.8	0.962
73, 76	T2-iso/hyper-intensity (n, %)	49, 67.1	39, 75	10, 47.6	**0.048**

Abbreviations: R-SRL, patients resistant to somatostatin receptor ligands; S-SRL, patients responsive to somatostatin receptor ligands; IGF-I, insulin-like growth factor I; ULN, upper limit of normal; r-GH, random growth hormone levels; PRL, prolactin.

**Table 3:** Bivariate logistic regression analyses on the most promising predictors of resistance to fg-SRL, identified by descriptive statistics.

Model	Data Available (n, %)	AUC (95% CI)	Variable	Coefficient	OR (95% CI)	*p*-Value
1	94, 97.9	0.62 (0.51–0.71)	Age (years)	−0.04	0.96 (0.93–0.99)	**0.035**
	
			IGF-I (ng/mL)			>0.1
2	73, 76	0.64 (0.52–0.75)	T2-iso/hyperintensity	1.19	3.3 (1.14–9.54)	**0.027**
	
			Maximal diameter (mm)			>0.1
3	89, 92.7	0.7 (0.59–0.79)	Low-grade SSTR2 expression	1.52	4.58 (1.37–15.29)	**0.013**
			
			SG/intermediate pattern	0.97	2.65 (1.01-6.92)	**0.047**
4	73, 76	0.76 (0.64–0.85)	T2-iso/hyperintensity	1.17	3.24 (1.03–10.2)	**0.045**
			
			SG/intermediate pattern	1.71	5.56 (1.75–17.6)	**0.003**
5	42, 82.3	0.82 (0.67–0.92)	No post-surgical appreciable remnant	−3.09	0.045 (0.01–0.24)	**0.0003**
	
			Random GH (ng/mL)			>0.1

Abbreviations: AUC, area under the curve; CI, confidence interval; OR, odds ratio; SSTR2, somatostatin receptor type 2; SG, sparsely granulated; IGF-I, insulin-like growth factor I; GH, growth hormone.

**Table 4:** Univariate logistic regression analyses on predictors of resistance to fg-SRL, selected from those significantly associated with non-response to treatment in bivariate regression models.

**Variable**	**Data Available**	**Coefficient**	**OR (95% CI)**	***p*-Value**	**AUC (95% CI)**
Age (years)	96, 100% (63 R-SRL, 33 S-SRL)	−0.03	0.97 (0.93–1.0008)	0.056	0.6 (0.5–0.7)
Low-grade SSTR2 expression	89, 92.7% (61 R-SRL, 28 S-SRL)	1.43	4.17 (1.29–13.49)	**0.017**	0.63 (0.52–0.73)
T2-iso/hyper-intensity	73, 76% (52 R-SRL, 21 S-SRL)	1.19	3.3 (1.14–9.54)	**0.027**	0.64 (0.52–0.75)
SG/intermediate CAM5.2 pattern	96, 100% (63 R-SRL, 33 S-SRL)	0.8	2.24 (0.95–5.28)	0.066	0.6 (0.49–0.7)

Abbreviations: OR, odds ratio; CI, confidence interval; AUC, area under the curve; R-SRL, patients resistant to somatostatin receptor ligands; S-SRL, patients responsive to somatostatin receptor ligands; SSTR2, somatostatin receptor type 2; SG, sparsely granulated.

**Table 5:** Histological and molecular features of operated GH-secreting tumors.

Variable	Data Available	All Patients	R-SRL	S-SRL	*p*-Value
Ki-67 ≥ 3% (n, %)	94, 97.9%	24, 25.5%	17, 27.9%	7, 21.2%	0.646
p53 ≥ 1% (n, %)	61, 63.5%	14, 23%	10, 22.7%	4, 23.5%	0.785
CAM5.2 pattern - DG (n, %) - SG/intermediate (n, %)	96, 100%	40, 41.7% 56, 58.3%	22, 34.9% 41, 65.1%	18, 54.5% 15, 45.4%	0.102
SSTR2 expression - 0–1 (n, %) - 2–3 (n, %)	89, 92.7%	29, 32.6% 60, 67.4%	25, 40.9% 36, 59%	4, 14.3% 24, 85.7%	**0.024**

Abbreviations: R-SRL, patients resistant to somatostatin receptor ligands; S-SRL, patients responsive to somatostatin receptor ligands; DG, densely granulated; SG, sparsely granulated; SSTR2, somatostatin receptor type 2.

## Data Availability

The datasets generated and analyzed during the current study are available from the corresponding author upon reasonable request.

## Supplementary Materials

The following supporting information can be downloaded at: https://www.mdpi.com/article/10.3390/jcm12010025/s1, Table S1: Available characteristics of the GH and IGF-I assays used at the various participating centers.
