# Supplementary material for: Resistance to Somatostatin Analogs in Italian Acromegaly Patients: The MISS Study
Source: J Clin Med. 2022 Dec 20;12(1):25. doi: 10.3390/jcm12010025 (PMC9821091; doi:10.3390/jcm12010025)
Supplement: Supplementary file 1 [file jcm-12-00025-s001.zip › jcm-2085313-supplementary.pdf]

Page 4 of 14
We added the acronym for prolactin

Page 5 of 14
We used italics for "*p*-value" as used throughout the text and proposed capital letter if "*P*-value" was the first word in the sentence.

Page 6 of 14
We removed the word "significantly"
We used the acronym PRL

Table 1
We moved "92, 95.8" from the third row to the second
We proposed capital letter for "*P*-value" in the column header

Table 2
We proposed capital letter for "*P*-value" in the column header

Table 3
We proposed capital letter for "*P*-value" in the column header
We corrected the punctuation

Table 4
We proposed capital letter for "*P*-value" in the column header

Table 5
We proposed capital letter for "*P*-value" in the column header

Page 10 of 14
We changed "for" to "to"

Page 11 of 14
We changed "3" to "three"
We corrected the Founding Information as follows: "Contribution and support agreement for non-profit clinical trials between the Department of Medical Sciences of the University of Turin and Italfarmaco S.p.A, date 18/5/2018"
